# Comprehensive analysis of POLE and POLD1 Gene Variations identifies cancer patients potentially benefit from immunotherapy in Chinese population

**DOI:** 10.1038/s41598-019-52414-z

**Published:** 2019-10-31

**Authors:** Jianfei Yao, Yuan Gong, Wei Zhao, Zhifeng Han, Shaohua Guo, Hongyi Liu, Xiumei Peng, Wenhua Xiao, Yuemin Li, Shiying Dang, Guifeng Liu, Lifeng Li, Tanxiao Huang, Shifu Chen, Lele Song

**Affiliations:** 1 HaploX Biotechnology, Co., Ltd, Shenzhen, P.R. China; 2 Department of Gastroenterology, the Chinese PLA General Hospital, Beijing, P.R. China; 3 Department of Thoracic Surgery, Sino-Japanese Friendship Hospital, Jilin University, Changchun, Jilin Province P.R. China; 4 Department of General Surgery, the Chinese PLA General Hospital, Beijing, P.R. China; 5 Department of Oncology, the Fourth Medical Center of the Chinese PLA General Hospital, Beijing, P.R. China; 6 Department of Radiotherapy, the Eighth Medical Center of the Chinese PLA General Hospital, Beijing, P.R. China

**Keywords:** Tumour biomarkers, Predictive markers

## Abstract

POLE/POLD1 gene variants have been suggested as potential markers for immunotherapy due to their significant association with the tumor mutational burden (TMB), an effective indicator for response prediction in immunotherapy. However, the correlation of POLE/POLD1 variants with MSI, MMR, TMB, MMR-related and key driver gene mutations needs to be defined to support patient recruitment and therapeutic effect assessment in immunotherapy. 1,392 Chinese cancer patients were recruited, and the correlation of POLE/POLD1 variants with existing immunotherapeutic markers and cancer pathways was investigated. A next-generation sequencing panel including 605 cancer-related genes was used for variant sequencing. It was found that the frequency of POLE variants was not statistically different from that in COSMIC database, while the frequency of POLD1 variants was significantly higher in lung cancer. c.857 C > G and c.2091dupC were potential high frequency variants in Chinese cancer patients. Patients carrying POLE damaging variants were significantly younger than POLE/POLD1 WT patients. Patients carrying POLE/POLD1 damaging variants exhibited significantly higher TMB and frequency of MMR gene variants than POLE/POLD1 WT patients. Patients with POLE damaging variants also exhibited significantly higher frequency of driver gene variants than POLE/POLD1 WT patients. Further analysis showed that POLE damaging variants may affect the cancer development through MMR, TGFβ and RTK/RAS/RAF signaling pathways, and POLD1 through MMR pathways. In conclusion, this study identified key characteristics and regions of POLE/POLD1 genes that correlates with TMB, MMR gene mutations and key driver gene mutations, and provided theoretical and practical basis for patient selection based on POLE/POLD1 gene status in immunotherapy.

## Introduction

In recent years, immunotherapy targeting PD-1/PD-L1 has achieved encouraging therapeutic effects in many cancers^1–3^. However, the overall response rate for unselected cancer patients is only approximately 20%. A series of markers for immunotherapy, including PD-1/PD-L1 expression, microsatellite instability (MSI), mismatch repair (MMR) and tumor mutation burden (TMB), can be used to enrich populations that are sensitive to PD-1/PD-L1-targeted immunotherapy. For example, Pembrolizumab has achieved satisfactory therapeutic effects in patients with high PD-L1 expression, MSI-High or dMMR^4^. Nivolumab has also performed well in cancer patients with MSI-H, dMMR and high TMB^5–7^. In addition to these commonly used markers, there are also some genetic variants closely related to the known markers of immunotherapy. Some studies reported that KRAS/TP53 mutation was positively correlated with PD-L1 expression, and therefore was closely related to immunotherapeutic effect^8,9^. Others reported that the POLE and POLD1 gene damaging variants were associated with higher TMB^10–14^, and therefore they may also be relevant to the efficacy of immunotherapy.

The precise ability in DNA replication and proofreading is a key factor to ensure high-fidelity inheritance and preventing tumor formation. The components encoded by POLE and POLD1 genes are major catalytic and proofreading subunits of the Polε and Polδ enzyme complexes, respectively. The Polε gene directs the synthesis of the leading strand of DNA, and the Polδ gene directs the synthesis of the lagging strand of DNA, and both of them have 3′-5′ exonuclease activity and polymerase activity. The 3′-5′ exonuclease activity of POLE and POLD1 increases the accuracy of DNA replication by 100 times, and plays crucial roles in DNA replication and repair^15–17^. It has been reported that damaging variations in the Polε and Polδ genes can affect genomic stability and directly lead to mutation increase and subsequent tumor formation. For example, it was reported that exonuclease region variants of POLE and POLD1 genes were closely correlated with the mutation formation and characteristics in endometrial cancer and colorectal cancer^10,18–20^.

There are some case reports showing that cancer patients with POLE or POLD1 damaging variants can benefit from immunotherapy. The types of cancers included NSCLC, endometrial cancer, glioblastoma, and colorectal cancer^11,12,21,22^. Since these reports suggested that patients’ immunotherapeutic benefits are associated with high TMB, which correlated with POLE and POLD1 damaging variants, it is necessary to study the characteristics of POLE and POLD1 variants and their relationship with current immunotherapy markers. In this study, we investigated the characteristics of POLE and POLD1 variants in Chinese population, and explored the relationship between POLE/POLD1 variants and age, MSI, TMB, MMR-related gene mutations and driver gene mutations. Our study provided a theoretical and practical basis for patient selection for immunotherapy in Chinese cancer patients with POLE and POLD1 variants.

## Methods and Materials

### Ethics approval and consent to participate

All experiment plans and protocols for the study were submitted to the ethics/licensing committees of the named participating hospitals for review and approval before the start of the clinical study, and were approved by the corresponding committees of hospitals, including the Chinese PLA General Hospital, the Sino-Japanese Friendship Hospital of Jilin University, the Fourth Medical Center of the Chinese PLA General Hospital, and the Eighth Medical Center of the Chinese PLA General Hospital. Confirmation of approval for clinical studies was received from all named institutional review board or ethics committee before the start of the clinical study. All subjects signed the informed consent before tissue or blood collection, and they were informed of the usage of samples and the test results. Informed consent was collected and obtained from all subjects before the start of the clinical study. All experiments, methods, procedures and personnel training were carried out in accordance with relevant guidelines and regulations of participating hospitals and laboratories.

### Study design, patients and samples

**Figure 1:**
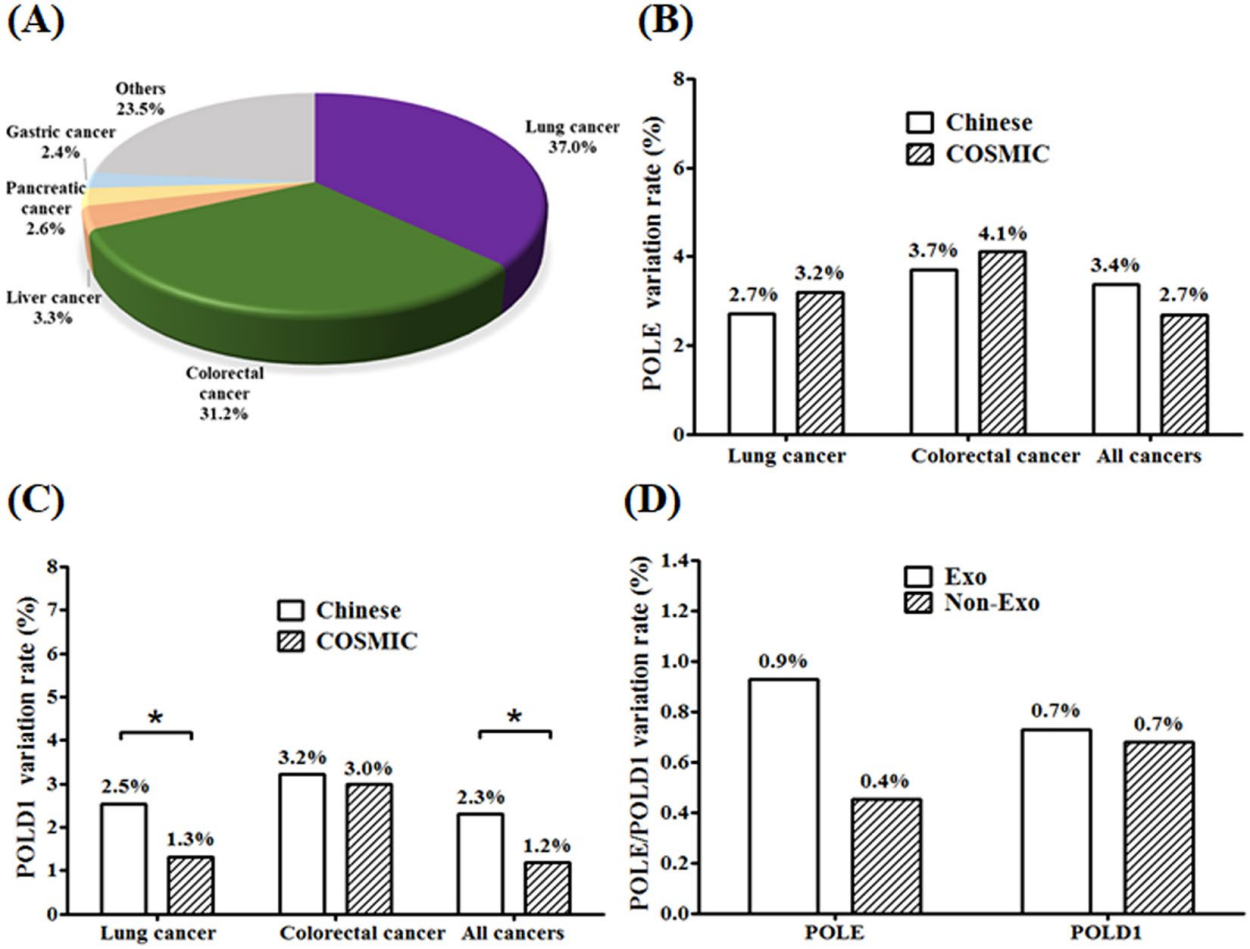
Distribution of cancer types and comparison of POLE/POLD1 variation rate with COSMIC database. The distribution and percentage of main cancer types involved in this study are shown in panel A. The comparison of variation rate in lung cancer, colorectal cancer and all cancers between this study and the COSMIC database is shown for POLE (panel B) and POLD1 (panel C), respectively. The variation rate of exonuclease region and non-exonuclease region normalized to region length for POLE and POLD1 is shown in panel D. *P < 0.05.

The POLE and POLD1 study was designed and implemented in four Chinese hospitals using the next-generation sequencing (NGS) methods. The study was designed to include as many cancer patients as possible regardless of cancer type, as long as the tissue or blood samples were available for NGS. As a result, the biggest cancer types in this study, based on natural distribution of cancer patients available for NGS test in Chinese cancer hospitals, were lung cancer and colorectal cancer. Clinical status of patients was determined before the collection of samples, including fresh surgical samples, needle biopsy samples, formalin-fix paraffin-embedded (FFPE) samples or blood which were obtained from all subjects who were confirmed with diagnosis of cancer. All technicians were blinded to the clinical information of subjects. A total of 1392 subjects were recruited in this study, including 516 lung cancer patients, 434 colorectal cancer patients, 46 liver carcinoma patients, 36 pancreatic cancer patients, 33 gastric cancer patients and 327 patients with other cancers (Fig. 1A, Supplementary Table 1). The classification of all conditions was based on diagnosis from imaging examinations and subsequent pathological examinations. None of the subjects received chemotherapy, radiotherapy, targeted therapy or immunotherapy before tissue or blood samples were collected. In order to balance the huge difference in the numbers between patients with or without POLE/POLD1 mutations, we used a randomized method to select the WT subjects by assigning a random number for each of 1392 patients with the function of RAND in Excel, and 78 subjects were selected to match the number of patients with POLE/POLD1 variants (Table 1).

**Table 1:** Age, gender and MS status for subjects with POLE/POLD1 variants or POLE/POLD1 WT in this study.

Clinicopathologic Factor	Number of patients (%)								P
	POLE&POLD1 WT	POLE Neu Var	P	POLD1 Neu Var	P	POLE Dam Var	P	POLD1 Dam Var	
**Age**									
≥60	47 (60.3)	11 (57.9)	0.524	8 (44.4)	0.448	7 (25.9)	0.002	8 (57.1)	0.93
<60	31 (39.7)	5 (26.3)		8 (44.4)		20 (74.1)		5 (35.7)	
≥50	62 (79.5)	13 (68.4)	1	13 (72.2)	1	15 (55.6)	0.015	10 (71.4)	1
<50	16 (20.5)	3 (15.8)		3 (16.7)		12 (44.4)		3 (21.4)	
≥40	74 (94.9)	16 (84.2)	1	15 (83.3)	1	21 (77.8)	0.026	11 (78.6)	0.2
<40	4 (5.1)	0 (0)		1 (5.6)		6 (22.2)		2 (14.3)	
unkown	0 (0)	3 (15.8)		2 (11.1)		0 (0)		1 (7.1)	
**Gender**									
female	36 (46.2)	6 (31.6)	0.526	7 (38.9)	0.86	11 (40.7)	0.626	5 (35.7)	0.77
male	42 (53.8)	10 (52.6)		9 (50.0)		16 (59.3)		7 (50.0)	
unkown	0 (0)	3 (15.8)		2 (11.1)		0 (0)		2 (14.3)	
**MS status**									
MSS	50 (64.1)	6 (31.6)	0.124	5 (27.8)	0.357	15 (55.6)	1	4 (28.6)	0
MSI	3 (3.8)	2 (10.5)		1 (5.6)		1 (3.7)		4 (28.6)	
unkown	25 (32.1)	11 (57.9)		12 (66.7)		11 (40.7)		6 (42.9)	

WT = wild type; Neu = neutral; Var = variant; Dam = damaging; MS = microsatellite; MSS = microsatellite stable; MSI = microsatellite instable.

### Sample preparation, targeted NGS and data processing

For the FFPE samples, ten 5 μm tumor slices were used for DNA extraction using the QIAamp DNA FFPE Kit (QIAGEN, Valencia, CA, USA) following the manufacturer’s instructions. For blood samples, 10 ml blood were collected in tubes containing EDTA and centrifuged at 1,600 × g for 10 min at 4 °C within 2 h of collection. The peripheral blood lymphocyte (PBL) debris was stored at −20 °C until further use. The supernatants were further centrifuged at 10,000 × g for 10 min at 4 °C, and plasma was harvested and stored at −80 °C until further use. DNA from fresh tissue samples was extracted using the EasyPure® Genomic DNA Kit (Beijing TransGen Biotech, Beijing, China). DNA from PBLs was extracted using the RelaxGene Blood DNA system (Tiangen Biotech Co., Ltd., Beijing, China) and ctDNA was extracted from at least 2 ml plasma using the QIAamp Circulating Nucleic Acid kit (Qiagen, Inc., Valencia, CA, USA) according to the manufacturers’ instructions. DNA was quantified with the Qubit 2.0 Fluorometer and the Qubit dsDNA HS assay kit (Thermo Fisher Scientific, Inc., Waltham, MA, USA) according to manufacturer’s instructions^23^. The integrity and fragment size are detected with Agilent 4200 TapeStation system (Agilent Technologies, USA). After DNA fragmentation, size selection of the DNA fragments was performed using Ampure XP beads (Beckman Coulter, Inc., Brea, CA, USA). DNA fragments and ctDNA were used for library construction using the KAPA Library Preparation kit (Kapa Biosystems, Inc., Wilmington, MA, USA) according to the manufacturer’s protocol. End repair and 3′-end A-tailing were performed following DNA fragmentation. Notably, T-tailed adapters were used and a 3′dA overhang was added enzymatically onto the fragmented DNA sample. The ligated fragments were then amplified using 1x KAPA HiFi Hot Start Ready mix (Kapa Biosystems, Inc.) and Pre-LM-PCR Oligos (Kapa Biosystems, Inc.) in 50 µl reactions, and 7–8 PCR cycles were performed depending on the amount of DNA input. Hybridization-based target enrichment was carried out with HaploX pan-cancer gene panel (605 cancer-relevant genes, HaploX Biotechnology, gene list was provided in Supplementary Table 2). Library fragment size was determined using the Agilent 4200 TapeStation system (Agilent Technologies, USA) after library construction. The library was quantified using qPCR before sequencing. DNA sequencing was then performed on the Illumina Novaseq. 6000 system according to the manufacturer’s recommendations.

Sequencing data were filtered by fastp version 0.18.0 (https://github.com/OpenGene/fastp)^24^, and aligned to the hg19 genome (GRch37) using Burrows Wheeler Aligner version 0.7.15 r1140 using default settings^25^. Data quality control was then carried on, and data which meet the following criteria were chosen for subsequent analysis: the ratio of remaining data filtered by fastq in raw data is ≥85%; the proportion of Q30 bases is ≥85%; the ratio of reads on the reference genome is ≥85%; target region coverage ≥98%; average sequencing depth in tissues is ≥500×; average sequencing depth in blood cfDNA is ≥1500×. The Gencore version 0.12.0 (https://github.com/OpenGene/gencore) was used to remove duplicate reads^26^. Pileup files for properly paired reads with mapping quality ≥60 were generated using Samtools version 0.1.19 (http://www.htslib.org/)^27^. Somatic variants were determined using VarScan2 version 2.3.8 (http://varscan.sourceforge.net/)^28^. The called somatic variants need to meet the following criteria: the read depth at a position is ≥20x; the variant allele frequency (VAF) is ≥2% for tissue DNA and ≥0.05% for cfDNA from blood; somatic-P value ≤0.01; strand filter ≥1. Allele frequencies were calculated for Q30 bases. The copy number variation was detected by CNVkit version 0.9.3 (https://github.com/etal/cnvkit)^29^.

### Interpretation of damaging variation, calculation of TMB and definition of driver genes

Damaging variants or neutral variants were defined and predicted based on the records and rules of COSMIC database [25]. If no pathogenicity information were found in COSMIC, then the online tools of CONDEL (http://bbglab.irbbarcelona.org/fannsdb/help/condel.html) was used to predict the pathogenicity of certain sites. TMB was calculated by dividing the total number of tissue non-synonymous SNP and Indel variations (allele frequency ≥5%) by the size of the coding region of the 605 panel^30–32^. Driver genes refer to the driver genes defined by the NCCN guidelines and those predicted by the intogen website (https://www.intogen.org/). Driver gene included in the 605 gene panel were sequenced, analyzed and the variations were called, including the full exon regions of ALK, ARID1A, APC, ATM, BRAF, BRCA1, BRCA2, CDH4, CTNNB1, EGFR, FBXW7, FGFR1, FGFR2, FGFR3, GNAS, HER2, HGF, KIT, KRAS, MET, PDGFRA, RB1, RET, ROS1, MLH1, MLH3, MSH2, MSH3, MSH6, NRAS, NTRK1, NTRK3 and PDGFRA, PIK3CA, PMS1, SMAD2, SMAD4, SMARCA4,TGFBR2, TP53.

### The definition for MSS and MSI

Based on the National Cancer Institute (NCI) recommendation, five microsatellite sites, including NR21, NR24, NR27, BAT25 and BAT26 were applied in MSI determination. If length variation of a microsatellite site is found in the tumor sample that is not found in the corresponding normal sample shown by capillary electrophoresis, the microsatellite marker is termed altered^33^. The tumors were classified as follows: if one of the five sites is altered, the sample is determined as MSI-low, and if two or more of the five sites are altered, the sample is determined as MSI-high, while if none of the five microsatellite sites is altered, the sample is determined as microsatellite stable (MSS)^34^.

### Statistics and data analysis

Statistical analysis was performed and figures were plotted with Graphpad Prism 5.0 software (GraphPad Software, Inc, La Jolla, CA 92037, USA). Student t-test was performed when two groups were compared, and ANOVA and post hoc tests were performed when three or more groups were compared. Chi-square test and Fisher test were performed when rate or percentage was compared for significance. Mutation spectrum figures were made with the R software (https://www.r-project.org/) ‘*’ represents P < 0.05 (significant), ‘**’ represents P < 0.01 (highly significant), and ‘***’ represents P < 0.001 (very highly significant).

## Results

### Characteristics of the somatic POLE and POLD1 variants in Chinese population

1,392 Chinese cancer patients were recruited in this study, including lung cancer (37.0%), colorectal cancer (31.2%), liver cancer (3.3%), pancreatic cancer (2.6%), gastric cancer (2.4%) and other cancer patients (23.5%) (Fig. 1A, Supplementary Table 1). The frequency of variations in POLE in lung cancer, colorectal cancer, and all cancer patients was 2.7% (14/516), 3.7% (16/434), and 3.4% (47/1392), respectively. The frequency of variation in POLD1 in lung cancer, colorectal cancer, and all cancer patients was 2.5% (13/516), 3.2% (14/434), and 2.3% (32/1392), respectively. The overall frequency of POLE variation in lung cancer, colorectal cancer, and all cancers in COSMIC database was 3.2% (132/4138), 4.1% (93/2290), and 2.7% (1503/55824), respectively, exhibiting no statistical significance to that in Chinese cancer patients (Fig. 1B). The overall frequency of POLD1 mutations in lung cancer, colorectal cancer, and all cancers in COSMIC database were 1.3% (56/4232), 3.0% (56/1853), and 1.2% (572/48271), respectively. The frequency of variation of POLD1 in lung cancer patients and all cancer patients in this study was significantly higher than that of the COSMIC database, while no such difference was found with colorectal cancer (Fig. 1C).

**Figure 2:**
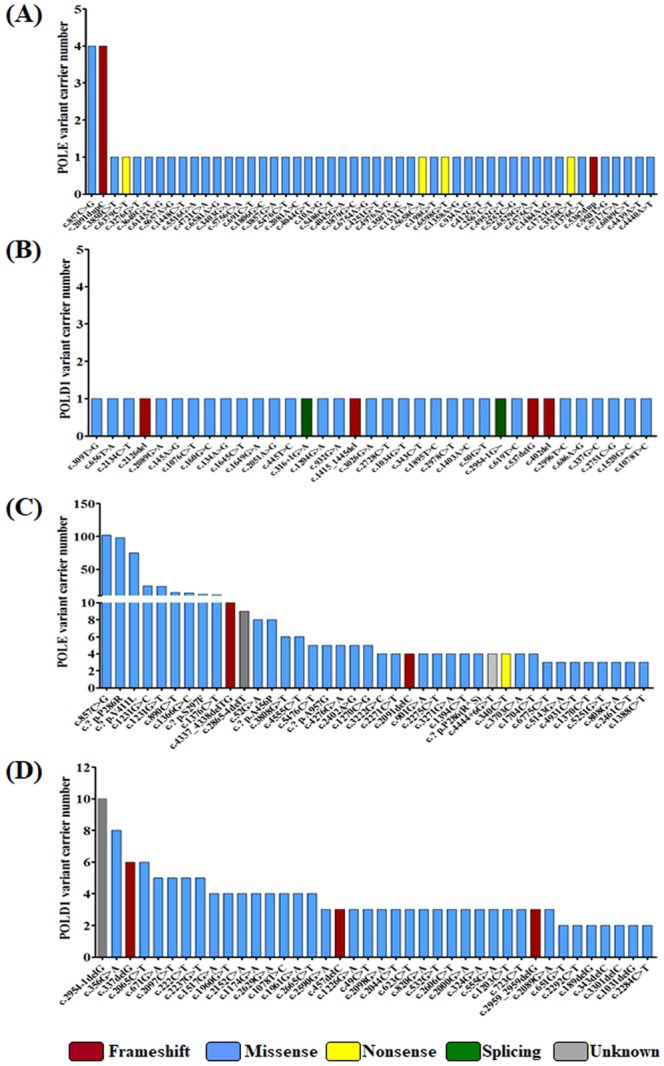
The frequency and distribution of somatic POLE and POLD1 variants in Chinese cancer patients, and a comparison with COSMIC database. All variants for POLE and POLD1 in this study are shown in panel A and panel B, respectively, including 50 POLE variants (from 56 subjects, including 6 repeated sites) and 35 POLD1 variants. The frequency and distribution of the top 40 variants of POLE and POLD1 from COSMIC database are shown for comparison in panel C and panel D, respectively. The types of variants are indicated by different colors.

**Figure 3:**
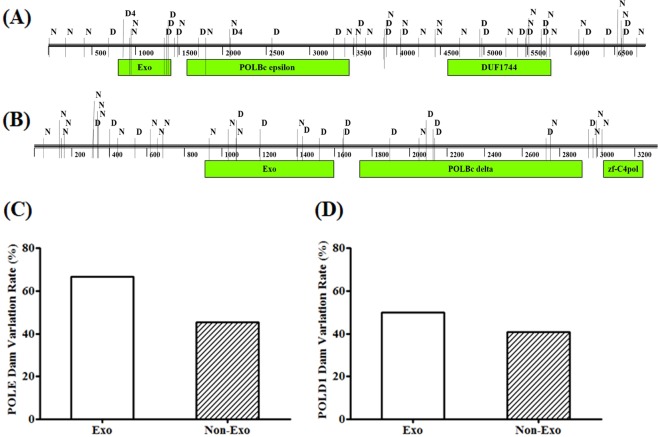
The distribution of damaging and neutral variants along the full-length POLE or POLD1. The distribution of variants of POLE and POLD1 is shown in (**A**,**B**), respectively. Green bars indicate the functional domains. The percentage of damaging variants in exonuclease or non-exonuclease region for POLE and POLD1 is shown in (**C**,**D**), respectively. No significant difference was found in the variant percentage between the two regions for POLE and POLD1. D = damaging; N = Neutral; Exo = exonuclease region; non-Exo = non-exonuclease region. Numbers indicate the number of cases for the same site.

The variations of POLE and POLD1 are distributed throughout the coding region of the gene. Following the exon length normalization, the variation frequency of POLE in the Exo or the Non-Exo region was 0.9% and 0.4%, respectively, and the variation frequency of POLD1 in the Exo region and the Non-Exo region was approximately 0.7% (Fig. 1D). The main variants of POLE and POLD1 are missense mutations. The potential highest frequency variations in POLE were c.857 C > G (p.P286R) and c.2091dupC (p.F699Vfs*11), and both were identified in 4 patients (Fig. 2A). c.857 C > G (p.P286R) also ranked the highest in COSMIC database (Fig. 2C). In contrast, only one record for c.2091dupC (p.F699Vfs*11) were found in COSMIC. The c.2091dupC (p.F699Vfs*11) variation could be a characteristic high frequency variation in the Chinese population. Due to the limited number of POLD1 variations, no hot spot variation in the POLD1 was identified, while c.2954-1delG and 356 G > A appeared to be the most frequent variations in COSMIC database (Fig. 2B,D). The distribution and position of the predicted damaging and neutral variations of the POLE and POLD1 gene are shown in Fig. 3A,B, respectively, with annotations of key functional domains (green bars). Further analysis showed that there was no statistically significant difference in the proportion of damaging variations between Exo and Non-Exo regions in POLE and POLD1 genes, ranging from 40% to 67% (Fig. 3C,D).

### POLE damaging mutations were correlated with younger cancer onset, and POLD1 damaging variants were correlated with higher MSI

**Figure 4:**
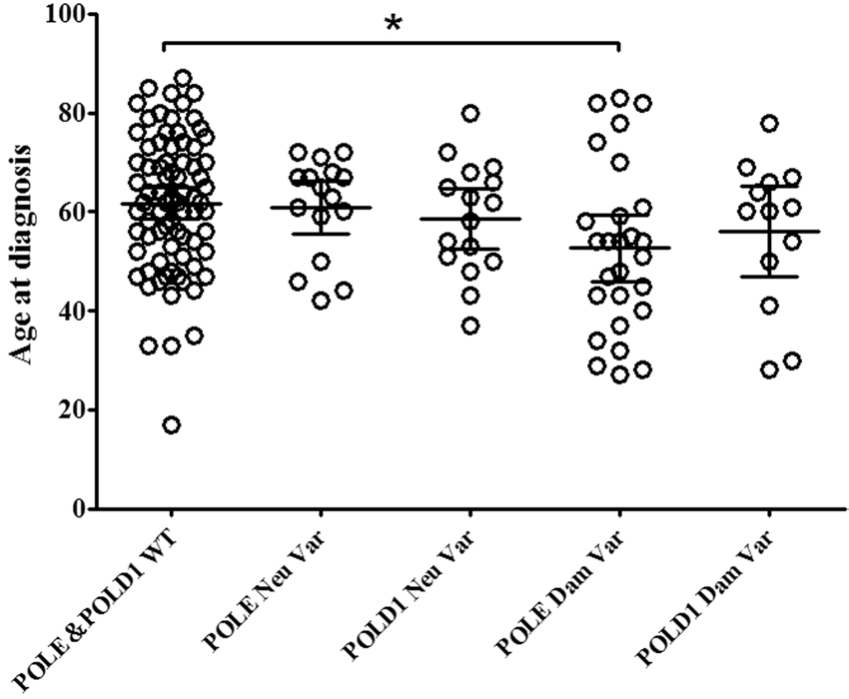
Age distribution for wild type, neutral or damaging POLE or POLD1 variants. Significant difference in age distribution is found between the WT and the POLE damaging variant groups. WT = wild type; Var = variant; Dam = damaging; Neu = neutral. *p < 0.05.

The age at diagnosis of the POLE damaging variant group (average 52.67, 95% CI: 45.95–59.39) was significantly lower than that of the POLE&POLD1 WT (WT) group (average 61.73, 95% CI: 58.63–64.84) (Fig. 4), while no statistical difference in the age of patients with neutral POLE variants compared with the POLE&POLD1 WT group, and no significant difference in the age of the POLD1 damaging or neutral variant group compared with the POLE&POLD1 WT group. In the POLE damaging mutation group, the proportion of patients younger than 40, 50 and 60 was 22.2%, 44.4% and 74.1%, respectively, which were significantly higher than that in the corresponding age groups of the POLE&POLD1 WT patients (Table 1). Further studies found that the frequency of MSI in the POLD1 damaging mutation group was significantly higher than that in the POLE&POLD1 WT group, while the frequency of MSI in the POLE damaging mutation group was not significantly different from that in the POLE&POLD1 WT group. Meanwhile, there was no statistical difference in the ratio of male to female in each groups compared with the POLE&POLD1 WT group (Table 1).

### POLE/POLD1 damaging variants led to higher TMB in Chinese cancer patients

**Figure 5:**
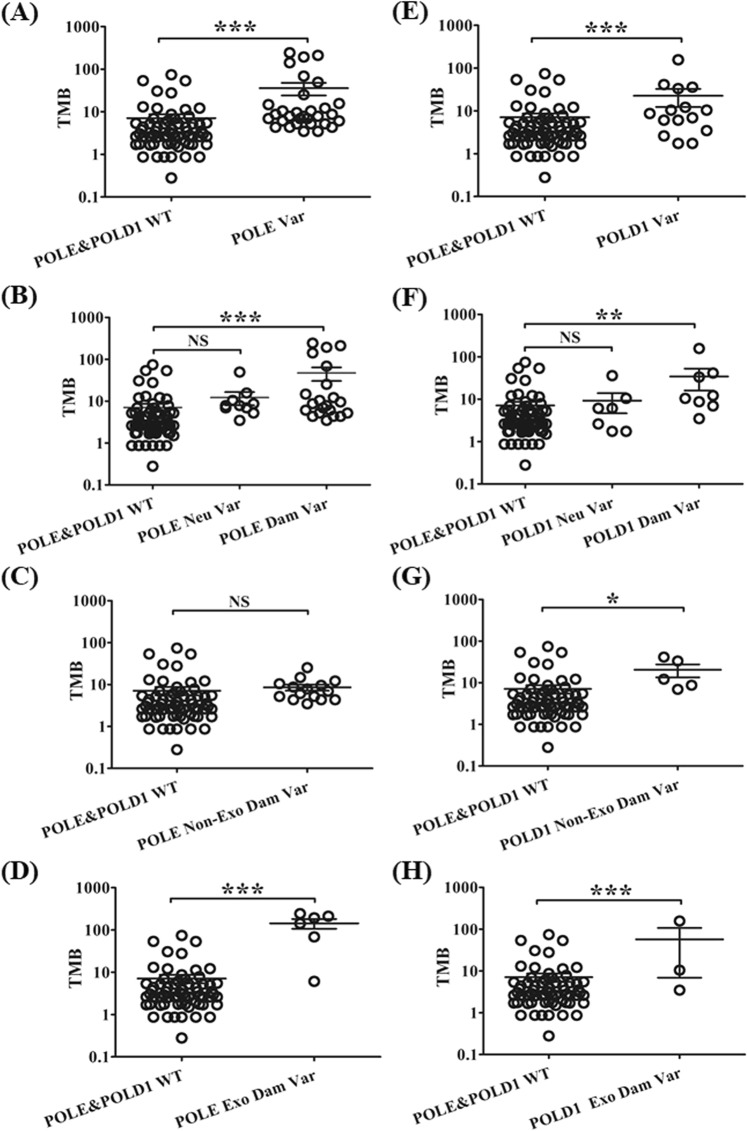
The relationship between POLE and POLD1 variants and TMB in Chinese cancer patients. The comparison between POLE/POLD1 WT and each variant group for POLE is shown in A-D, and the comparison between POLE/POLD1 WT and each variant group for POLD1 is shown in E-H. WT = wild type; Var = variant; Dam = damaging; Exo = exonuclease region; Non-Exo = non-exonuclease region; Neu = neutral. *P < 0.05; **P < 0.01; ***P < 0.001.

The TMB of patients with POLE or POLD1 variants was significantly higher than that of POLE&POLD1 WT group (Fig. 5A,E). The TMB of patients with POLE or POLD1 damaging variants was significantly higher than that of POLE&POLD1 WT group, while the TMB of patients with POLE and POLD1 neutral variants exhibited no significant difference to that of the POLE&POLD1 WT group (Fig. 5B,F). Further analysis showed that the TMB of the POLE Exo region damaging variant group, the POLD1 Exo and the Non-Exo region damaging variant group was significantly higher than that of the POLE&POLD1 WT group, while the TMB of the POLE Non-Exo region damaging variant group showed no differences to that of the POLE&POLD1 WT group (Fig. 5C,D,G,H). These results suggest that damaging variants of the POLE Exo region and POLD1 were correlated with elevated TMB.

### POLE damaging variants led to higher mutation level in MMR-related genes and driver genes, and POLD1 damaging variants led to higher mutation level in MMR-related genes

**Figure 6:**
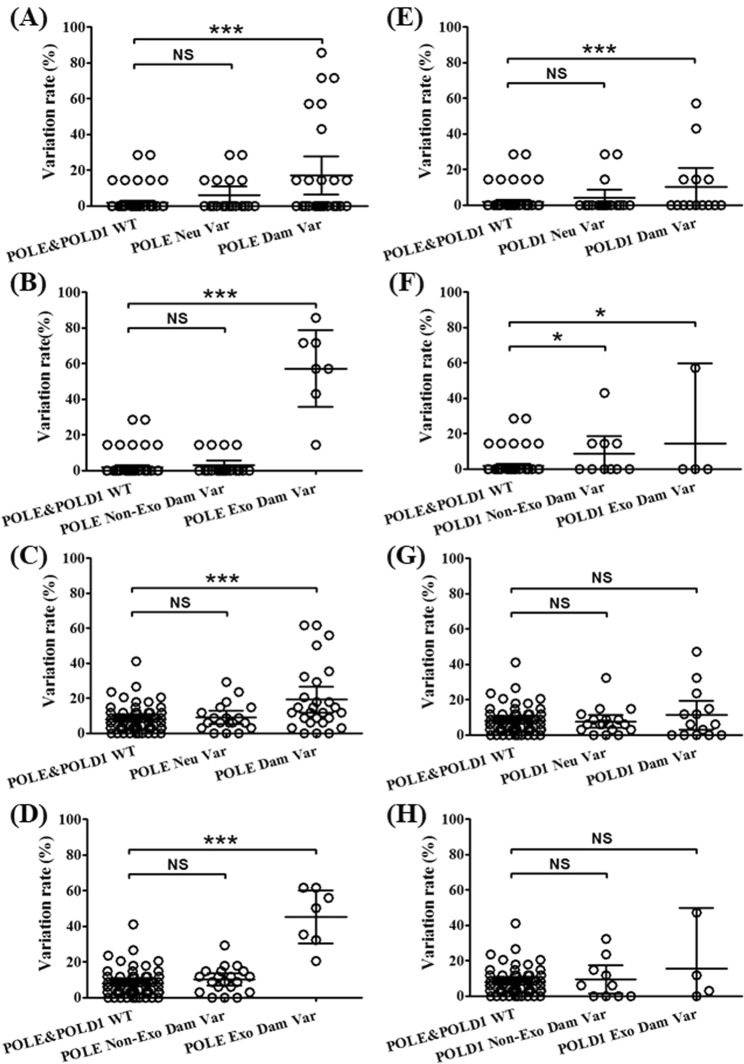
The relationship between POLE and POLD1 variants and the mutation rate of MMR-related genes or driver genes. The MMR-related mutation rate in POLE variant groups is shown in A and B, and the diver gene mutation rate in POLE variant groups is shown in C and D. The MMR-related mutation rate in POLD1 variant groups is shown in E and F, and the diver gene mutation rate in POLD1 variant groups is shown in G and H. WT = wild type; Var = variant; Dam = damaging; Exo = exonuclease region; Non-Exo = non-exonuclease region; Neu = neutral. *P < 0.05; **P < 0.01; ***P < 0.001.

In order to match with the number of patients with POLE/POLD1 variants, 78 patients were randomly selected from the POLE&POLD1 wile type (WT) group for analysis in this study. We explored the relationship between POLE/POLD1 variation groups and the mutation frequency of MMR-related genes or the driver genes. Patients with POLE damaging variants exhibited significantly higher mutation frequencies of MMR-related or driver genes than those of POLE&POLD1 WT. Further analysis revealed that it was the damaging variants in POLE Exo region, not the non-Exo region that were correlated with high frequencies of MMR-related (Fig. 6A,B) or driver gene mutations (Fig. 6C,D). In contrast, patients with POLD1 damaging mutations exhibited higher frequency of MMR-related gene mutations than that of the POLE&POLD1 WT group (Fig. 6E,F), while no such difference were found with driver gene mutations (Fig. 6G,H). It was also found that both Exo and Non-Exo region damaging variants in POLD1 increased the mutation frequency of MMR-related genes (Fig. 6F).

**Figure 7:**
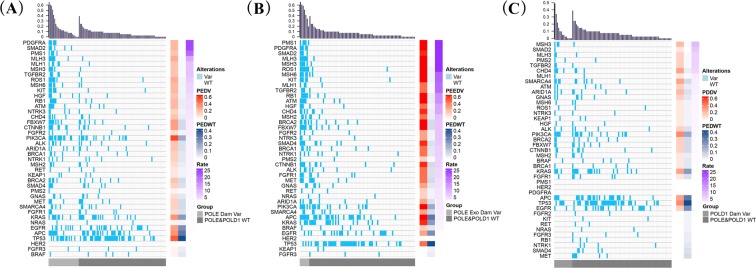
The mutation spectrum of MMR-related genes and driver genes in patients with POLE and POLD1 variants. The mutation spectrum of cancers with 27 POLE damaging variants is shown in (**A**) and compared with that of the POLE/POLD1 WT group (78 cases, randomly selected). The mutation spectrum of cancers with 7 POLE exonuclease region damaging variants is shown in (**B**) and compared with that of the POLE/POLD1 WT group (78 cases, randomly selected). The mutation spectrum of cancers with 14 POLD1 damaging variants is shown in (**C**) and compared with that of the POLE/POLD1 WT group (78 cases, randomly selected). Names of the MMR-related and driver genes are list to the left of the spectrum panels. Histograms above the spectrum panels show the mutation rate for each case, ranked from highest to lowest in each group. Grey bars under the spectrum panels indicates the different groups, with light grey bars representing the POLE or POLD1 damaging variant groups, and the dark grey bars representing the POLE/POLD1 WT group. Red bars on the right represent the mutation rate for each listed gene in patients with POLE or POLD1 damaging variants, and the darkness stands for the level of mutation rate. Blue bars on the right represent gene mutation rate for POLE/POLD1 WT group, with darkness standing for the level of mutation rate. Purple bars on the right represent the ratio of mutation rate between POLD/POLD1 damaging variant group and the WT group, ranked from highest to lowest.

The details of the high-frequency MMR and driver gene mutations in the POLE damaging mutation group, the POLE Exo damaging mutation group and the POLD1 damaging variant group is shown in Table 2. In order to clarify the quantitative relationship between POLE/POLD1 damaging mutations and the frequency of MMR or driver gene mutations, we further quantified the mutation frequency ratio between patients with POLE/POLD1 damaging mutations and those with POLE&POLD1 WT. Figure 7 shows the mutation frequency ratio between POLE damaging variation group and POLE&POLD1 WT group (Fig. 7A), or between POLE Exo region damaging variation group and POLE&POLD1 WT group (Fig. 7B), or between POLD1 damaging variation group and POLE&POLD1 WT group (Fig. 7C). It was found that the mutation frequency of MMR-related genes in the POLE damaging variation group was much higher than that of POLE&POLD1 WT group. The ratio of variation frequency of PMS1, MLH3, MSH3, MLH1, MSH6 and PMS2 was 19.52, 11.80, 8.55, 8.55, 6.52 and 2.06 respectively, between the two groups (Fig. 7A, Table 3). The ratio is even higher when only the Exo region damaging variation is compared, and the ratio of variation frequency of PMS1, MLH3, MSH3, MLH1, MSH6 and PMS2 was 72.43, 38.00, 31.74, 19.22, 20.32 and 6.70, respectively (Fig. 7B, Table 3).

**Table 2:** Key driver gene mutation rate in cancer patients with POLE or POLD1 damaging variants.

Gene Name	POLE Dam Var	Gene Name	POLE Exo Dam Var	Gene Name	POLD1 Dam Var
PIK3CA	51.9%	APC	100.0%	PIK3CA	35.7%
TP53	48.2%	ROS1	100.0%	TP53	35.7%
APC	33.3%	MLH3	85.7%	APC	28.6%
KRAS	33.3%	PIK3CA	71.4%	KRAS	28.6%
CTNNB1	29.6%	FBXW7	71.4%	CHD4	28.6%
EGFR	29.6%	ATM	71.4%	SMARCA4	28.6%
FBXW7	29.6%	KIT	71.4%	MSH3	21.4%
MLH3	25.9%	MSH6	71.4%	ARID1A	21.4%
ROS1	25.9%	PDGFRA	71.4%	MLH3	14.3%
ATM	22.2%	RB1	71.4%	FBXW7	14.3%
KIT	22.2%	BRCA2	71.4%	ATM	14.3%
MSH6	22.2%	MSH3	71.4%	BRCA2	14.3%
PDGFRA	22.2%	PMS1	71.4%	CTNNB1	14.3%
RB1	22.2%	KRAS	57.1%	ALK	14.3%
SMAD2	22.2%	CTNNB1	57.1%	TGFBR2	14.3%
ALK	18.5%	SMAD2	57.1%	EGFR	14.3%
ARID1A	18.5%	CHD4	57.1%	GNAS	14.3%
BRCA2	18.5%	SMAD4	57.1%	PMS2	14.3%
CHD4	18.5%	TP53	42.9%		
HGF	18.5%	ALK	42.9%		
MLH1	18.5%	HGF	42.9%		
MSH3	18.5%	MLH1	42.9%		
PMS1	18.5%	TGFBR2	42.9%		
TGFBR2	18.5%	BRCA1	42.9%		
BRCA1	14.8%	MET	42.9%		
MET	14.8%	NTRK1	42.9%		
NTRK1	14.8%	FGFR1	42.9%		
NTRK3	14.8%	MSH2	42.9%		
SMAD4	14.8%	EGFR	28.6%		
SMARCA4	14.8%	ARID1A	28.6%		
FGFR1	11.1%	NTRK3	28.6%		
FGFR2	11.1%	SMARCA4	28.6%		
GNAS	11.1%	FGFR2	28.6%		
MSH2	11.1%	GNAS	28.6%		
		RET	14.3%		
		BRAF	14.3%		
		NRAS	14.3%		
		PMS2	14.3%		

Dam = damaging; Exo = exonuclease region; Var = variants;

**Table 3:** Mutation rate and mutation ratio for key driver genes when comparing POLE/POLD1 damaging variant groups with the WT group.

Gene Name	PEDWT*	PEDV*	Ratio (PEDV/PEDWT)	PEEDV*	Ratio (PEEDV/PEDWT)	PDDV*	Ratio (PDDV/PEDWT)
SMAD2	0.0%	22.2%	23.22	57.1%	58.14	7.1%	8.14
PDGFRA	0.0%	22.2%	23.22	71.4%	72.43	0.0%	1.00
PMS1	0.0%	18.5%	19.52	71.4%	72.43	0.0%	1.00
MLH3	1.3%	25.9%	11.80	85.7%	38.00	14.3%	6.70
MSH3	1.3%	18.5%	8.55	71.4%	31.74	21.4%	9.83
TGFBR2	1.3%	18.5%	8.55	42.9%	19.22	14.3%	6.70
MLH1	1.3%	18.5%	8.55	42.9%	19.22	7.1%	3.57
ROS1	2.6%	25.9%	7.55	100.0%	28.34	7.1%	2.28
MSH6	2.6%	22.2%	6.52	71.4%	20.32	7.1%	2.28
KIT	2.6%	22.2%	6.52	71.4%	20.32	0.0%	0.28
HGF	2.6%	18.5%	5.48	42.9%	12.31	7.1%	2.28
ATM	3.8%	22.2%	4.79	71.4%	14.95	14.3%	3.15
RB1	3.8%	22.2%	4.79	71.4%	14.95	0.0%	0.21
NTRK3	2.6%	14.8%	4.44	28.6%	8.30	7.1%	2.28
CHD4	3.8%	18.5%	4.03	57.1%	12.00	28.6%	6.10
FBXW7	7.7%	29.6%	3.52	71.4%	8.33	14.3%	1.76
CTNNB1	7.7%	29.6%	3.52	57.1%	6.69	14.3%	1.76
FGFR2	2.6%	11.1%	3.40	28.6%	8.30	0.0%	0.28
PIK3CA	17.9%	51.9%	2.79	71.4%	3.82	35.7%	1.94
ARID1A	6.4%	18.5%	2.63	28.6%	3.99	21.4%	3.03
ALK	6.4%	18.5%	2.63	42.9%	5.92	14.3%	2.06
BRCA1	5.1%	14.8%	2.58	42.9%	7.16	7.1%	1.33
NTRK1	5.1%	14.8%	2.58	42.9%	7.16	0.0%	0.16
MSH2	3.8%	11.1%	2.50	42.9%	9.05	7.1%	1.68
KEAP1	2.6%	7.4%	2.36	0.0%	0.28	7.1%	2.28
RET	2.6%	7.4%	2.36	14.3%	4.29	0.0%	0.28
BRCA2	7.7%	18.5%	2.25	71.4%	8.33	14.3%	1.76
SMAD4	6.4%	14.8%	2.13	57.1%	7.85	0.0%	0.13
PMS2	1.3%	3.7%	2.06	14.3%	6.70	14.3%	6.70
GNAS	5.1%	11.1%	1.98	28.6%	4.83	14.3%	2.49
SMARCA4	7.7%	14.8%	1.82	28.6%	3.40	28.6%	3.40
MET	7.7%	14.8%	1.82	42.9%	5.05	0.0%	0.12
FGFR1	6.4%	11.1%	1.63	42.9%	5.92	7.1%	1.10
KRAS	21.8%	33.3%	1.51	57.1%	2.55	28.6%	1.30
NRAS	2.6%	3.7%	1.32	14.3%	4.29	0.0%	0.28
EGFR	24.4%	29.6%	1.21	28.6%	1.17	14.3%	0.60
APC	29.5%	33.3%	1.13	100.0%	3.31	28.6%	0.97
TP53	47.4%	48.1%	1.01	42.9%	0.91	35.7%	0.76
HER2	0.0%	0.0%	1.00	0.0%	1.00	0.0%	1.00
FGFR3	3.8%	3.7%	0.97	0.0%	0.21	0.0%	0.21
BRAF	5.1%	3.7%	0.77	14.3%	2.49	7.1%	1.33

PEDWT = POLE/POLD wide type; PEDV = POLE damaging variant; PEEDV = POLE Exo region damaging variant; PDDV = POLD1 damaging variant. *: 1% was added to both the numerator and the denominator when the original values of one of them or both were 0% in ratio calculation.

The ratio of TGFβ signaling pathway-related genes, including SMAD2, TGFβR2 and SMAD4, was 23.22, 8.55 and 2.13, respectively, when POLE damaging variation group is compared with the WT group, and was 58.14, 19.22 and 7.85, respectively, when POLE Exo region damaging variation group is compared with the WT group. In the RTK/RAS/RAF pathway-related genes, the ratio for PDGFRα, KIT, NTRK3, NTRK1, FGFR2, and RET gene mutation was 23.22, 6.52, 4.44, 2.58, 3.40 and 2.36, respectively, between the POLE damaging variation group and the WT group (Fig. 7A, Table 3), and was 72.43, 20.32, 8.30, 7.16, 8.30 and 4.29, respectively, between the POLE Exo region damaging variation group and the WT group (Fig. 7B, Table 3). In contrast, the ratio of some other driver genes, including KRAS, NRAS, EGFR, HER2, BRAF and FGFR3 was below 2 in POLE damaging variation group, and was 2.55, 4.29, 1,17, 1.00 2.49, 0.21, respectively, in POLE Exo region damaging variation group (Fig. 7A,B), suggesting minor effect by POLE damaging mutations.

The ratio of MMR-related genes between POLD1 damaging variation group and the POLE&POLD1 WT group, including MSH3, MLH3, PMS2, MLH1, MSH6 and PMS1, was 9.83, 6.70, 6.70, 3.57, 2.28, 1, respectively (Fig. 7C, Table 3). The ratio between the two groups in TGFβ signaling pathway-related genes, SMAD2, TGFβR2 and SMAD4, was 8.14, 6.70 and 0.13, respectively. The ratio of most of the RTK/RAS/RAF pathway genes was below 2 between the two groups (Fig. 7C, Table 3).

All the above analysis suggests that POLE damaging mutations, especially the damaging mutations in the POLE Exo region, play a key role in the mutation frequency of MMR-related genes and driver genes, and the most affected pathways include TGFβ and RTK/RAS/RAF signaling pathways.

## Discussion

### Main characteristics of POLE and POLD1 variants reflected the uniqueness of Chinese cancer patients

In this study, we observed three features related to POLE and POLD1 variants. First, the frequency of POLD1 mutations in Chinese lung cancer patients was higher than that recorded in COSMIC^35^. This difference may be explained by race discrepancy or the distinct distribution of various pathological types of lung cancer, while direct evidence is still needed. It was reported that patients with lung adenocarcinoma in the Asian population exhibited a higher frequency of EGFR mutations than that of the Western population^36^, and women with no smoking history exhibited even high frequency of EGFR mutations. It can be speculated that the reason for the higher frequency of POLD1 mutations in Chinese lung cancer patients may also be related to the race or gender relevant distinct pathological characteristics different from Western populations. Therefore, the cause of the frequency difference of POLD1 variants in Chinese lung cancer patients and its significance for immunotherapy deserve further investigation.

Secondly, the hot spot mutations of POLE gene in Chinese lung cancer were c.857 C > G (p.P286R) and c.2091dupC (p.F699Vfs*11), while the hot spot mutation recorded by COSMIC were c.857 C > G (p.P286R) and c.1231 G > C/T (p.V411L). c.857 C > G (p.P286R) appeared to be the top hot spot mutation in both Chinese population and the COSMIC database, while c.2091dupC (p.F699Vfs*11) could be a specific hot spot mutation in Chinese population. It is a frameshift mutation that causes a stop codon insertion, which may result in a truncated, immature or non-functional protein. Since it is located in the DNA polymerase type-B epsilon subfamily catalytic domain and is interpreted as a damaging mutation based on its position and type, it may cause disruption of the key polymerase domain and compromise the whole protein function. In contrast, there is only one record in the COSMIC database for this mutation in a colorectal cancer cell line. Therefore, no systematic study of the function of this mutation has been carried out. The function and the significance of c.2091dupC in Chinese population still need further investigation.

Thirdly, in this study, patients with POLE or POLD1 damaging mutations had a significantly higher incidence of cancer than those with POLE&POLD1 WT in age groups less than 40, 50 or 60 years old. Previous studies have shown that POLE mutations are associated with an earlier age of onset. A study in the US population showed that the proportion of women younger than 60 years old was significantly elevated in endometrial cancer patients carrying POLE variants^37^. Our study indicated that the proportion of patients younger than 40 years old with POLE damaging mutations was significantly higher than that of POLE&POLD1 WT patients, suggesting that the effects of POLE damaging mutations in Chinese population may be more significant. The Chinese population may be more sensitive to the damaging mutations of POLE and the age of onset was further advanced.

### The relationship between POLE and POLD1 variants with MSI, TMB, MMR-related gene and driver gene mutations

In this study, the pathogenicity of the POLE/POLD1 gene was determined based on previous reference and the interpretation by COSMIC and CONDEL^38^. No significant correlation between the neutral variations of POLE/POLD1 and the TMB, MSI, MMR-related genes or driver gene mutations has been identified, indicating that the interpretation of pathogenicity are in accordance with expectations. This interpretation method can be used for predicting the pathogenicity of mutations.

Previous studies reported controversial findings in the correlation between POLE variants and MSS/MSI. Some found that POLE mutations were associated with higher MSS frequency^18,38^, while some revealed no significant correlation between POLE mutations and MSS/MSI^37^. Others reported that mutations in the POLE and POLD1 genes are associated with higher MSI^39^. Our study indicated that there was no significant correlation between POLE damaging variation and MSS/MSI in the Chinese population, while the damaging variation of POLD1 was associated with higher MSI. The reason for the above discrepancy is still not clear, while the following a few factors could affect the interpretation. First, the definition of damaging or neutral mutations is not unified among studies. Secondly, the interpretation of MSI and MSS may be different. Thirdly, the impact of POLE/POLD1 on MSI/MSS may not be similar in different populations.

Reports on the relationship between POLE/POLD1 variants and the TMB appeared to be more consistent among studies. Existing evidence have shown that TMB was higher in cancer patients with POLE and POLD1 variants^10–14^. However, most of studies focused on mutations in the Exo region, or did not distinguish between the Exo and Non-Exo regions. Our study investigated the TMB in terms of variants in both Exo and Non-Exo regions and found that TMB was higher in patients with damaging mutations in Exo region of POLE, and TMB was higher in patients with damaging mutations in Exo and Non-Exo regions of POLD1. These observations indicated that the effect of POLE gene on TMB is closely related to the function of Exo region. The abnormal function of this region may lead to abnormal DNA replication and correction, resulting in higher TMB. In contrast, the effect of POLD1 on TMB is not confined to the Exo region, and all damaging mutations can lead to an increased TMB. The divergent effects of POLE and POLD1 on TMB possibly reflect the differential functions of the two genes.

Our study also showed that POLE damaging mutations may affect TMB by influencing the frequency of MMR-related gene mutations and driver gene mutations, while POLD1 damaging mutations may affect the TMB by influencing part of MMR-related gene pathways and MSI. The effects of POLD1 on MMR-related mutations or pathways appeared to be less than that of the POLE, and generally had no significant effect on the frequency of variations of driver genes. This observation reflects the difference in pathways that POLE and POLD1 mutations affect the somatic mutations. Since the exonuclease regions of POLE and POLD1 are highly homologous (23% identity, 37% similarity), the exonuclease function of the POLE and POLD1 genes may be similar. However, there are also some differences in function between the two genes, in which POLE is responsible for the synthesis of the leading strand and POLD1 is responsible for the synthesis of the lagging strand during DNA replication. This may partially explain the different effects of POLE and POLD1 on MMR-related genes, MSI and TMB, while the exact mechanism is still to be explored.

### Conclusions and future perspectives

Our study clarified the relationship between POLE/POLD1 mutations and MSI and TMB in Chinese cancer patients. Since MSI and TMB are immunotherapy-related markers, this study demonstrates that patients with damaging variants of the Exo region of the POLE and the Exo and Non-Exo regions of POLD1 are potential population for immunotherapy. Patients with damaging mutations in POLE, especially those with damaging mutations in the POLE Exo region, had higher percentage of MMR-related and cancer-related driver gene mutations. Therefore, it can be speculated that the combination of immunotherapeutic drugs and certain pathway-targeted drugs may be a future direction for patients with damaging mutations in POLE. Clinical trials have already started. For example, the immunotherapeutic drug pembrolizumab was used in combination with lenvatinib, a targeted drug that inhibits the activity of VEGFR, FGFR, PDGFRα, KIT and Ret, demonstrated promising anti-tumor effect in patients with renal cell carcinoma or endometrial cancer^40^.

## Supplementary information

Supplementary Table 1

Supplementary Table 2

## Data Availability

The datasets used and/or analysed during the current study are available from the corresponding author on reasonable request.
